# A need for new tools for prevention of malaria in pregnancy

**DOI:** 10.1371/journal.pmed.1004729

**Published:** 2025-09-19

**Authors:** Makoto Saito, Rose McGready

**Affiliations:** 1 Centre for Tropical Medicine and Global Health, Nuffield Department of Medicine, University of Oxford, Oxford, United Kingdom; 2 WorldWide Antimalarial Resistance Network (WWARN) and Infectious Diseases Data Observatory (IDDO), Oxford, United Kingdom; 3 Shoklo Malaria Research Unit, Mahidol-Oxford Tropical Medicine Research Unit, Faculty of Tropical Medicine, Mahidol University, Mae Ramat, Thailand

## Abstract

Intermittent preventive treatment in pregnancy (IPTp) with sulfadoxine–pyrimethamine (SP) is a chemoprevention strategy against malaria in pregnancy. A recent *PLOS Medicine* study highlights the need for better understanding of the mechanism by which IPTp-SP reduces low birthweight, as well as novel measures to prevent malaria earlier in gestation.

Malaria is the most common parasitic disease in humans with over 250 million cases every year [1], affecting small children and pregnant women particularly. In 2023, an estimated 36 million pregnant women in sub-Saharan Africa were at risk of malaria and one-third had malaria infections [1]. Malaria parasites infect red blood cells, and *Plasmodium falciparum*, the most pathogenic species among the four species that primarily infect humans, can invade the placenta. In addition to maternal anemia, placental malaria causes placental insufficiency, leading to small-for-gestational-age (SGA), preterm birth, and fetal loss [2]. These harmful effects are evident even when the burden of parasitemia is below levels detectable by rapid diagnostic tests (RDT) or microscopy [3], highlighting the need to prevent malaria infection in the first place.

Intermittent preventive treatment in pregnancy (IPTp) is one of the chemoprevention strategies endorsed by the World Health Organization (WHO) since 1998 for moderate-to-high malaria endemic areas and is currently implemented in 38 countries [1]. The currently recommended regimen is three or more doses of the antimalarial sulfadoxine–pyrimethamine (SP) given at the full therapeutic dosage, repeated at least 4 weeks apart starting from the second trimester, regardless of gravidity. While low uptake (44% of women received three doses or more in 2023 [1]) is a practical challenge, another problem is the spread of high-level resistance to SP in falciparum parasites in east and southern Africa [4]. The most effective alternative antimalarial candidate has been monthly dihydroartemisinin–piperaquine (DP). Although IPTp-DP has been shown to have superior antimalarial efficacy to IPTp-SP, reducing the risk of clinical malaria episodes by two-thirds, placental malaria at delivery, and maternal anemia, many randomized controlled trials (RCTs) failed to show superiority in reducing the risk of low birth weight (LBW) [5].

On the contrary, the superior efficacy of IPTp-DP on malaria-related outcomes did not equate to superior anthropometric outcomes for the newborns (e.g., birth weight and infant growth by two-month old) or mothers (e.g., maternal gestational weight gain and middle-upper-arm-circumference at delivery) compared with IPTp-SP [5]. Similarly, IPTp-SP showed a reduced risk of LBW, maternal anemia, and preterm birth even in areas with higher-level SP resistance, where its antimalarial effects were almost completely lost [4]. The anthropometric benefits of SP, independent of its antimalarial effect, have been a mystery among malariologists. Several hypotheses have been proposed: SP’s broader antibacterial/antiparasitic spectrum, anti-inflammatory effects, and direct effects on nutritional absorption [4,5]. It is therefore quite natural to consider what would happen if SP and DP were given together.

In a recent *PLOS Medicine* study [6], Kakuru and colleagues conducted a large double-blinded RCT in Uganda, where high-level SP-resistant parasites harboring five or more mutations are prevalent [6]. In total, 2,757 HIV-uninfected pregnant women were enrolled and randomly allocated to one of the three monthly chemoprevention regimens: SP alone, DP alone or SP + DP. The key finding of this RCT is that a combination of SP + DP did not convey any additional benefits compared with SP or DP alone, including both malaria outcomes and the risk of composite adverse birth outcomes [6]. When fetal outcomes were assessed individually, the risks of SGA and LBW, the main components contributing to the composite outcome, were higher in SP + DP than in SP alone. In addition, the risk of preterm birth was lowest in the DP arm. The latter is consistent with subgroup analysis findings from an individual patient data meta-analysis that included only RCTs started at ≤20 weeks’ gestation [5]. Unexpectedly, some malaria outcomes in the SP + DP arm were slightly but statistically significantly inferior to those of DP alone.

The authors hypothesized at least two potential explanations: DP has a negative anthropometric effect, or DP and SP interact when co-administered [6]. Although the first explanation seems plausible, it does not explain the inferior malaria outcomes in SP + DP compared with DP, which a drug-drug interaction can better explain. A pharmacokinetic study nested within this RCT has revealed lower absorption of both SP and DP when they were given together [7]. This implies that DP and SP can be given on separate days to achieve their full potential.

Another interesting finding was that the maternal and fetal anthropometric benefits of SP were more pronounced or only observed in multigravida women, as was shown in a meta-analysis [5]. This is biologically plausible considering that the higher antimalarial effect of DP would benefit more primigravida women, whose risk of and impact from malaria infection is higher due to the lack of parity-dependent immunity against placental malaria. However, using different regimens depending on gravidity is logistically challenging, and there is also a potential risk of a rebound effect in the subsequent pregnancies due to the impairment of acquired parity-dependent immunity.

Regardless of the level of SP resistance, there are limitations to the current IPTp-SP policy. IPTp-SP is a monthly monotherapy, which will, based on its pharmacokinetics, neither cure existing parasitemia nor prevent new infections for a full month during pregnancy when the clearance of sulfadoxine is markedly increased [8]. IPTp-SP can suppress but not clear parasitemia; suppression can offer some benefit for primigravida women [5,6] but is not ideal as an intervention against malaria, which can be harmful even at a clinically undetectable level. The beneficial anthropometric effect of IPTp-SP must mostly come from its non-malarial effect, necessitating a proper preventive measure against malaria. Identification of the pathways SP exerts benefits may contribute to improved care and better use of SP, which is also an antibiotic. Additionally, IPTp-SP does not protect women in early gestation because of later antenatal care attendance and contraindication of SP, an antifolate combination, in the first trimester. As the placenta develops in the first 20 weeks, the adverse impact of malaria has already started and is probably most severe by the time IPTp-SP can be given. This RCT [6] revealed that 70% of women had parasitemia at enrollment (between 12 and 20 weeks); even very low parasitemia (more than 1000-times lower than the limit of detection by RDT) at the first antenatal care appointment was previously shown to be associated with lower birthweight [3]. More pre-conceptional studies, which start enrolling reproductive age women before gestation, would be beneficial to assess the impact of malaria and also the safety of antimalarials for treatment and prevention in early gestation. Preconception vaccines are also an alternative or an additional option to chemoprevention to protect from malaria infection, even before women become aware of their pregnancy. Vaccines targeting placental malaria are in the pipeline [9,10], as are non-placental-malaria vaccines [11], including the R21/Matrix-M malaria vaccine (NCT06080243), which are currently being tested specifically for women of reproductive age. Longevity of immunity is one of the key factors for the success of this approach.

The findings of the current study discourage the use of SP + DP as an alternative IPTp regimen. Biological explanations of the non-malarial effect of SP can be multi-factorial, and we now have another question about the apparent negative interactions between SP and DP, both of which require further study. A better understanding of these mechanisms, improvement in the uptake of IPTp, and measures to prevent malaria in early gestation, including preconception malaria vaccines, will all contribute to a better future in malaria-endemic areas by protecting future generations from the harm of malaria.

## Abbreviations

**:**
DP: dihydroartemisinin–piperaquine
IPTp: intermittent preventive treatment in pregnancy
LBW: low birth weight
RCTs: randomized controlled trials
RDT: rapid diagnostic tests
SGA: small-for-gestational-age
SP: sulfadoxine–pyrimethamine
WHO: World Health Organization
